# Understanding and predicting the functional consequences of missense mutations in BRCA1 and BRCA2

**DOI:** 10.1038/s41598-022-13508-3

**Published:** 2022-06-21

**Authors:** Raghad Aljarf, Mengyuan Shen, Douglas E. V. Pires, David B. Ascher

**Affiliations:** 1 Computational Biology and Clinical Informatics, Baker Heart and Diabetes Institute, Melbourne, VIC 3004 Australia; 2 Department of Biochemistry and Pharmacology, University of Melbourne, Melbourne, VIC 3010 Australia; 3 Systems and Computational Biology, Bio21 Institute, University of Melbourne, 30 Flemington Rd, Parkville, VIC 3052 Australia; 4 School of Computing and Information Systems, University of Melbourne, Melbourne, VIC 3053 Australia; 5 Department of Biochemistry, University of Cambridge, 80 Tennis Ct Rd, Cambridge, CB2 1GA UK

**Keywords:** Machine learning, Protein analysis, Medical genomics

## Abstract

*BRCA1* and *BRCA2* are tumour suppressor genes that play a critical role in maintaining genomic stability via the DNA repair mechanism. DNA repair defects caused by *BRCA1* and *BRCA2* missense variants increase the risk of developing breast and ovarian cancers. Accurate identification of these variants becomes clinically relevant, as means to guide personalized patient management and early detection. Next-generation sequencing efforts have significantly increased data availability but also the discovery of variants of uncertain significance that need interpretation. Experimental approaches used to measure the molecular consequences of these variants, however, are usually costly and time-consuming. Therefore, computational tools have emerged as faster alternatives for assisting in the interpretation of the clinical significance of newly discovered variants. To better understand and predict variant pathogenicity in *BRCA1* and *BRCA2*, various machine learning algorithms have been proposed, however presented limited performance. Here we present *BRCA1* and *BRCA2* gene-specific models and a generic model for quantifying the functional impacts of single-point missense variants in these genes. Across tenfold cross-validation, our final models achieved a Matthew's Correlation Coefficient (MCC) of up to 0.98 and comparable performance of up to 0.89 across independent, non-redundant blind tests, outperforming alternative approaches. We believe our predictive tool will be a valuable resource for providing insights into understanding and interpreting the functional consequences of missense variants in these genes and as a tool for guiding the interpretation of newly discovered variants and prioritizing mutations for experimental validation.

## Introduction

The breast cancer susceptibility gene 1 (*BRCA1*) and 2 (*BRCA2*) are tumour suppressor genes required in pathways responsible for repairing damaged DNA, transcriptional regulation, and maintaining genomic stability, as these are crucial mechanisms for cells to avoid apoptosis and chromosomal rearrangement^1^. Consequently, variants in these genes can predispose to multiple types of cancer^2^.

Genetic testing is widely used in the clinic to identify individuals at high risk of developing breast, ovarian, and other types of cancers and these individuals are frequently carriers of germline pathogenic variants that disrupt BRCA1 and BRCA2 DNA repair function^3^.

Germline variants in *BRCA1* and *BRCA2* contribute to 20–25% of hereditary breast and ovarian cancer^4^, while *BRCA1/2* somatic variants account for 5%–7% of ovarian cancers^5^ and up to 10% of breast cancers^6^. Individuals with *BRCA1/2* variants have an increased risk of developing both breast (84% increased risk) and ovarian (45% increased risk) cancers^6,7^. Pathogenic variants of *BRCA1/2* genes are associated with approximately 15–40% of hereditary breast cancers^8^. Individuals carrying *BRCA1* pathogenic variants have a 59% elevated risk of developing breast cancer and a 34% of developing ovarian cancer by age 70. In contrast, carriers of *BRCA2* pathogenic variants have a 51% risk of breast cancer and 11% risk of ovarian cancer at the age of 80 years^9^. Even though characterising a missense variant definitive pathogenicity status can better inform treatment, prevention and clinical management^4^, most missense variants identified by clinical genetic testing reported in public databases are listed as variants of uncertain significance (VUS)^10^. Thus, there is a need for accurate approaches to establish and predict variant pathogenicity and its impact on protein function.

Failure to precisely predict the consequences of missense variants in *BRCA1* and *BRCA2* genes confounds our understanding of sequencing data and impacts clinical care. To date, as only a limited number of missense variants have been functionally evaluated experimentally, the interpretation of variant pathogenicity has relied on applying in silico tools for predicting functional effects together with family-based data^11^.

Despite significant effort dedicated over the years to the development of accurate and general computational methods capable of identifying deleterious variants at genomic scale^12–15^, these have presented variable performance and reliability at a gene level^12,16–18^**.** In a particular example of *BRCA1/2*, Ernst et al*.* suggested after evaluating the performance of Align-GVGD^19,20^, SIFT^12^, PolyPhen-2^15^ and MutationTaster2^21^ on a set of well-characterized *BRCA1/2* variants, that the results obtained using in silico tools are insufficient to be applied as stand-alone evidence in clinical diagnostics^18^. Thus, the availability of experimentally characterized effects of variants would allow us to overcome this limitation by tailoring gene-specific predictive methods to uncover mutation-structure–function relationships.

With advances in bioinformatics and computational biology, several computational attempts have been made to explore the functional impacts of missense variants in *BRCA1* and *BRCA2* genes. Hart et al. implemented an in silico model BRCA-ML for understanding the functional impact of missense variants in *BRCA1* and *BRCA2* genes and VUS classification^11^. In addition, Arshad and colleagues investigated the structural and functional consequences of *BRCA1* variants on cellular mechanisms by applying well-established in silico approaches^22^*.* Finally, Ernst et al*.* evaluated the reliability of employing computational tools to predict the pathogenicity of *BRCA1* and *BRCA2* missense variants as the basis for clinical decision-making^18^. They analysed performance improvement effects by combining various in silico prediction approaches on a data set of well-characterized *BRCA1/2* missense variants in comparison to stand-alone tools.

Here we have developed a new machine learning method capable of accurately predicting the functional effect of missense variants in the *BRCA1* and *BRCA2* genes and implemented a computational saturation mutagenesis approach to classify all VUSs within these genes. We believe that our predictive models could be valuable for interpreting *BRCA1* and *BRCA2* variants and overcoming the challenge of classifying variants of uncertain significance, in addition to improving the clinical utility of genetic testing on these genes.

## Results

### Variant distribution in *BRCA1 *and *BRCA2*

**Figure 1:**
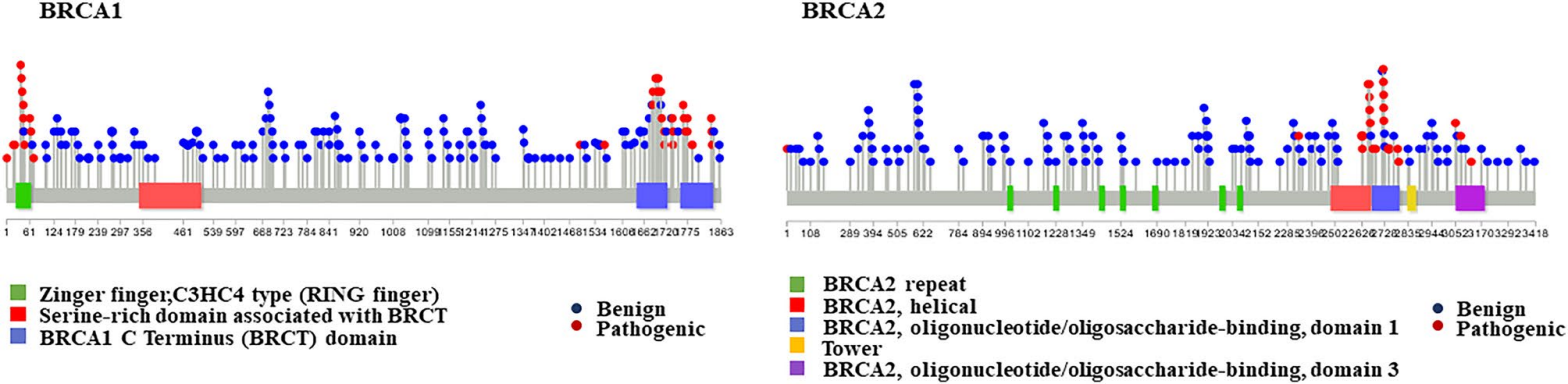
The distributions of BRCA1 and BRCA2 missense variants shown as lollipop plots. Benign and likely benign variants are represented by blue circles and red circles depict pathogenic and likely pathogenic variants. The mapped BRCA1 and BRCA2 missense variants are ranked for their impact at the protein level, particularly nonsynonymous missense variants.

In order to visualize the distributions of missense variants curated from ClinVar^10^ *BRCA1* and *BRCA2*, lollipop plots were generated and are depicted in Fig. 1. Most pathogenic variants observed were concentrated at well-known functional domains (BRCT and RING domains of BRCA1 and the DNA binding domain of BRCA2) of both genes, consistent with the previous findings^4^. Benign variants were uniformly distributed across both genes, covering 62% and 74% of BRCA1 and BRCA2 residues, respectively.

### Exploring the functional consequences of *BRCA* variants using statistical analysis and feature engineering

To distinguish between pathogenic and benign variants, we performed a qualitative analysis to investigate the relationship between different molecular properties with variant consequences. These included protein stability effects upon mutation, amino acid biophysical properties, effects on post-translational modifications and evolutionary conservation. A total of 197 features were calculated (Suppl. Table 1).

We conducted a Welch Two Sample t-test to identify features that could differentiate between the two classes, pathogenic and benign, in both *BRCA1* (Suppl. Figure 1) and *BRCA2* (Suppl. Figure 2) genes. For BRCA1, one of the most descriptive attributes was sequence conservation given as ConSurf scores^23^ (p < 2.2e-16), indicating that pathogenic variants tend to frequently occur in conserved regions, consequently leading to function impairment, in agreement with previous studies^24^. Other features highlighting the molecular differences between the two classes include amino acid physicochemical properties^25^. Particularly, features representing statistical potentials (KESO980102: *p* =  < 6.6e-06, MIRL960101: *p* =  < 1.1e-05 and *MIYT* 79,010: *p* =  < 1.1e-05) presented a significant difference between benign and pathogenic variants.

For BRCA2, highly discriminating features included sequence evolutionary conservation properties (PANTHER^26^ : *p* < 6.9e-13, ConSurf ^23^: *p* < 2.3e-15), suggesting that pathogenic variants tend to occur in conserved positions, as previously observed^24^. The stability analysis by SAAFEC-SEQ^27^ tool (p < 0.007) revealed that pathogenic variants were likely to be highly destabilizing, as shown before^24^. Furthermore, pathogenic variants displayed differential patterns in terms of amino acid physicochemical properties^25^ in comparison to benign variants (MUET020101: p < 0.003). These properties highlight the importance of considering a range of properties when assessing the functional impacts of variants on protein function.

**Figure 2:**
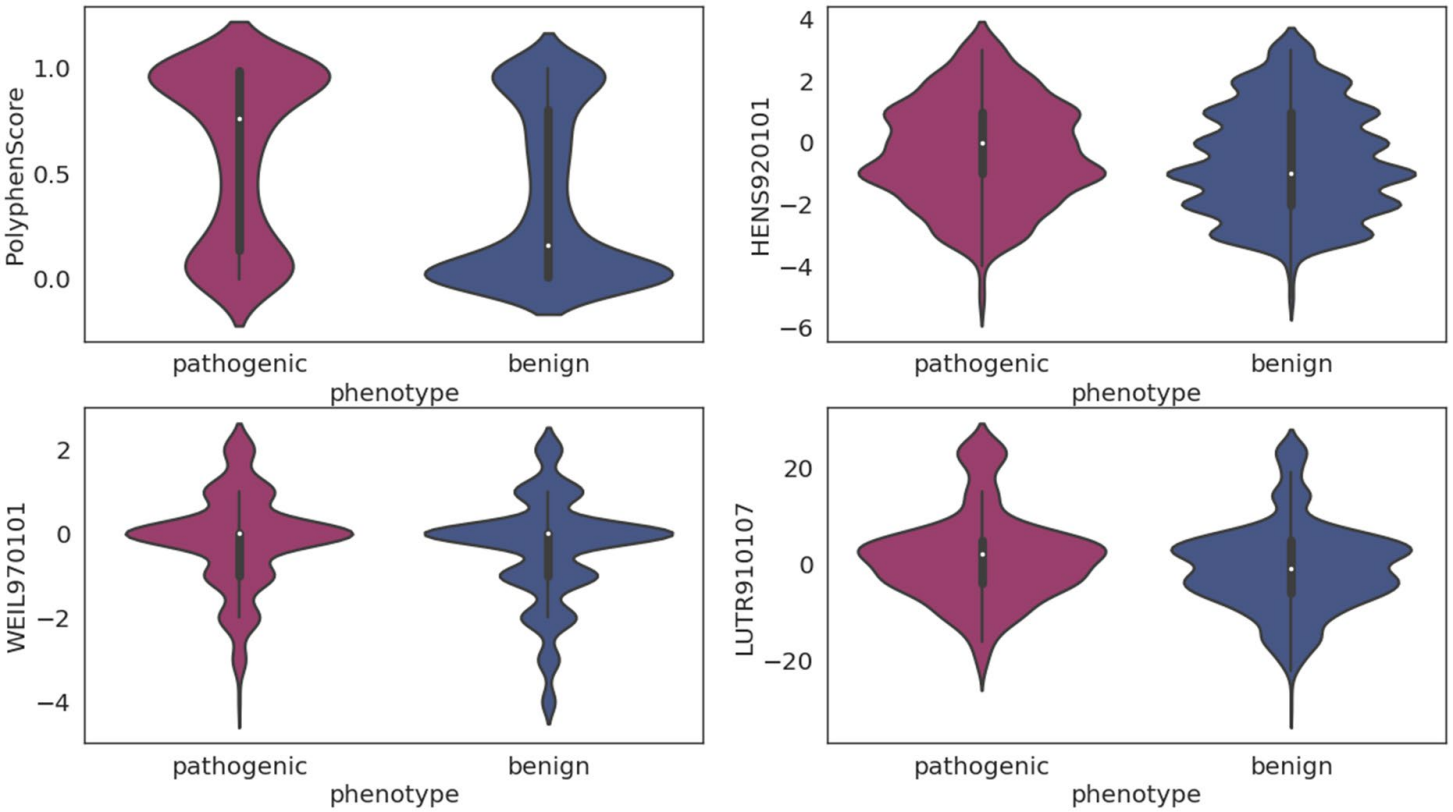
Distribution of the top discriminative features between the pathogenic and benign variants. Selected features incorporated sequence conservation and amino acids physicochemical properties. (PolyphenScore, HENS920101, WEIL970101 and LUTR910107). The selected features are significantly different between the two classes (*p* < 0.001). Statistical significance was measured using the Welch sample t-test.

For model optimization, Welch’s t-test was also conducted on all the features used in the final model (*BRCA1/2* combined) to provide biological insight into which distinct features characterize functional consequences of BRCA1 and BRCA2 upon single amino acid substitutions (Fig. 2). Among the most differentiating attributes were sequence-based conservation scores (PolyphenScore^28^) and amino acids physicochemical properties^22^: HENS920101 (represents the BLOSUM45 substitution matrix), WEIL970101 (represents amino acid comparative profiles) and LUTR910107 (represents mutation matrices for the various protein secondary structure classes^22^).

Following the elimination of redundant features, a greedy feature selection approach was performed, based on Matthew’s Correlation Coefficient (MCC). Our final optimal model included 15 features (Suppl. Table 2). These representative features of the varied classes considered involved conservative scores from Provean^29^ and PolyphenScore^28^. In addition, MetaSVM_score, MPC-rankscore, MutationTaster_score, ClinPred-score^28^, and physicochemical amino acid properties (AA-index)^22^ were included, as well as functional annotation scores from the AWESOME tool (predicting the effect of SNP on the level of the post-translational modification), including ubiquitination, acetylation and AWESOME Score^30^.

Notably, while AA-index^22^ provides a measure of numerical indices that represent different physicochemical properties of amino acids, only six of these features were selected by the greedy feature selection approach: MIYS990107 and THOP960101 are representations of the amino acid pair-wise contact potentials, while LUTR910107, HENS920101, WEIL970102, and WEIL970101 denote amino acid mutation matrices.

### Developing *BRCA1* and *BRCA2* gene-specific pathogenicity predictors

Different supervised learning algorithms were assessed to build gene-specific predictive models that can accurately identify pathogenic variants in *BRCA1* and *BRCA2* genes.

After greedy features selection, the best performing models were obtained using the Random Forest classifier (*n_estimators* = 300) for both genes. While for *BRCA1*-combined and *BRCA2*-combined (where pathogenic or likely pathogenic variants were grouped as pathogenic, and benign or likely benign variants were grouped as benign), the models with the best performances were the ensemble classifiers: Extra Trees (*n_estimators* = 300) and Gradient Boosting (*n_estimators* = 300), respectively.

**Figure 3:**
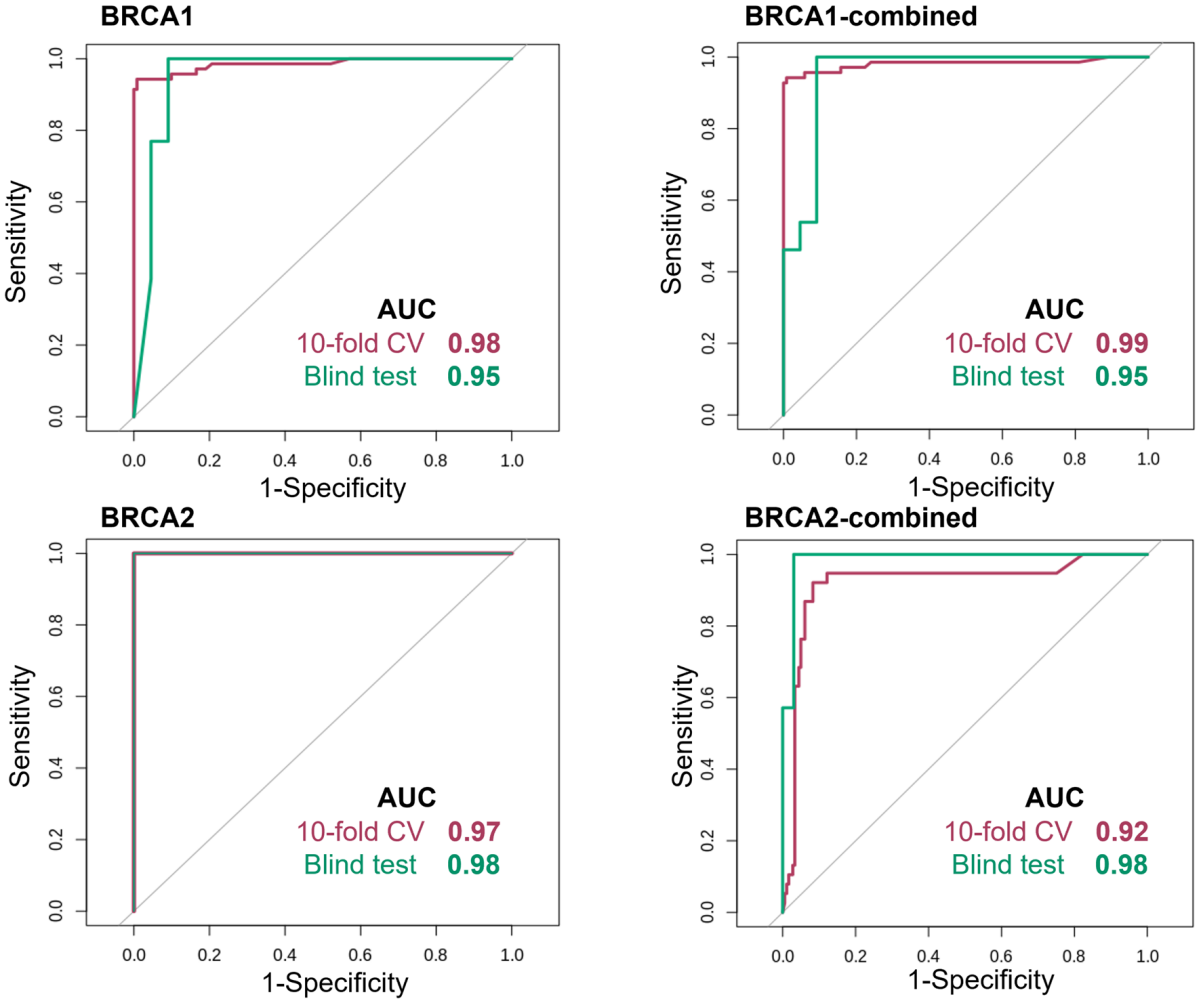
Receiver Operating Characteristic (ROC) curves for *BRCA1* (top) and *BRCA2* (bottom). Our predictive models accurately identified pathogenic variants with AUC > 0.92 on cross-validation and blind tests.

*BRCA1* and *BRCA2* gene-specific predictors achieved a range of Matthew’s Correlation Coefficient (MCC) varying from 0.89 to 0.98 across tenfold cross-validation and comparable performance of up to 0.89 across independent, non-redundant blind tests (Table 1). Furthermore, the final classification models achieved an AUC of up to 0.99 across tenfold cross-validation (Fig. 3) and comparable performance of up to 0.98 on independent, non-redundant blind tests.

**Table 1:** Comparative performance of *BRCA1/2* models across cross-validation and non-redundant blind test sets.

Model		MCC	Sensitivity	Specificity	F1 score	Precision	Accuracy
*BRCA1*-specific models							
*BRCA1*	CV	0.96	93%	98%	0.96	0.96	0.96
	test	0.89	92%	82%	0.86	0.87	0.86
*BRCA1*-combined	CV	0.96	94%	98%	0.97	0.97	0.97
	test	0.89	100%	91%	0.94	0.95	0.94
*BRCA1*-ENIGMA	CV	0.96	99%	99%	0.99	0.99	0.99
	test	0.82	92%	90%	0.93	0.94	0.92
*BRCA2*-specific models							
*BRCA2*	CV	0.98	96%	98%	0.98	0.98	0.98
	test	0.89	60%	100%	0.92	0.93	0.92
*BRCA2*-combined	CV	0.89	82%	93%	0.91	0.91	0.91
	test	0.83	86%	100%	0.97	0.98	0.97
*BRCA2*-ENIGMA	CV	0.95	99%	99%	0.99	0.99	0.99
	test	1.00	100%	100%	1.00	1.00	1.00
General model							
*BRCA1/2* general model	CV	0.91	96%	98%	0.96	0.96	0.96
	test	0.76	93%	100%	0.93	0.93	0.92

When comparing the predictions made by *BRCA1* and *BRCA2* gene-specific models, the *BRCA1* correctly identified 94 out of 97 pathogenic variants, and it wrongly classified 5 out of 150 benign missense variants. In contrast, we found that the *BRCA2* model was more accurate in classifying benign variants; it misclassified only one benign variant as pathogenic.

### Predicting the clinical significance of *BRCA1/2* variants using ENIGMA data

We build gene-specific predictive models to increase the reliability and evaluate the clinical impact of *BRCA1/2* missense variants. Therefore, we assessed different supervised learning algorithms to train (a binary classifier) and optimise the predictive capability of each model in classifying pathogenic variants in *BRCA1* and *BRCA2* genes using the Evidence-based Network for the Interpretation of Germline Mutant Alleles (ENIGMA) data^31^.

After greedy features selection, the models with the best performances were obtained using the ensemble classifier Gradient Boosting (n_estimators = 300) for both genes.

*BRCA1* and *BRCA2* gene-specific predictors performed a range of Matthew's Correlation Coefficient (MCC) ranging from 0.82 to 0.96 across tenfold cross-validation and comparative performance of up to 1.00 across independent, non-redundant blind tests (Table 1). Similarly, the final classification models achieved an AUC of up to 0.99 across tenfold cross-validation (Suppl. Figure 3) and equivalent performance of up to 1.00 on independent, non-redundant blind tests.

When looking closely at the predictions made by *BRCA1* and *BRCA2* (ENIGMA) gene-specific models, the *BRCA1* model accurately categorised 28 out of 29 pathogenic variants, and it incorrectly classified 1 out of 112 benign missense variants.

The misclassified variant, S1715R, is located in the BRCT domain of BRCA1 and has been previously revealed to disrupt BRCA1 interaction with Abraxas, BRIP1 and CtIP29^32^. It was also misclassified by other tools, including polyphen2^15^ and Align-GVGD^19,20^, highlighting that potentially including structural information into these predictions could further improve accuracy by capturing additional molecular consequences of variants.

### Developing a general *BRCA1/2* pathogenicity predictor

We investigated whether a general predictive tool could be developed to accurately classify pathogenic variants in *BRCA1* and *BRCA2* genes by combining all missense variants of both genes.

For the general *BRCA1/2* predictor (where all variants of both genes were combined), the final model with the best performance was obtained using the Random Forest classifier (*n_estimators* = 300). It achieved an accuracy of 0.96 on tenfold cross-validation, with an AUC of 0.96, MCC of 0.91, and precision of 0.96. This was comparable with the performance across the non-redundant blind test, achieving an AUC of 0.95, MCC of 0.76, and precision of 0.93, providing confidence in the generalizability of the final model (Table 1 and Suppl. Figure 4). When tested on all *BRCA1/2* variants in the training *BRCA1/2*-combined combined dataset (*n* = 406), our initial model’s performance in classifying pathogenic and benign variants was 91% and 98%, respectively.

Table 1 shows the performance of the classification models across tenfold cross-validation and blind test sets. The performance of our *BRCA1* and *BRCA2* gene-specific and general pathogenicity predictors was consistent on both tenfold cross-validation and blind test sets highlighting the robustness of the predictive models, and their capability of accurately differentiating between pathogenic and benign variants.

To better guide the interpretation of novel variants, we tested the applicability of our general model to predict the likelihood of pathogenicity of the Variants of Unknown Significance (VUS, *n* = 5716) in *BRCA1* and *BRCA2*. It was observed that our model predicted 13% of these as pathogenic and 87% as benign. According to the *BRCA1/2*- general model, the total proportion of all potential pathogenic variants in *BRCA1* and *BRCA2* is ~ 3% (891 out of 30,616). Nearly all of them are located in well-known functional domains (BRCT and RING domains of BRCA1 and the DNA binding domain of BRCA2), consistent with the previous findings^4^.

Interestingly, our model predicted the variant W31S located in the PALB2-binding domain of BRCA2 as pathogenic, which is consistent with a recent study finding^33^. The tryptophan residue at position 31 of BRCA2 is one of the essential residues for BRCA2 interaction with PALB2, as it is known to create a polar bridge with Ser1065 of PALB2^34^. Consequently, changing tryptophan to Serine would abolish BRCA2 binding to PALB2, as demonstrated previously in vitro^34,35^.

*BRCA1/2*- combined predicted scores for all possible single-nucleotide variation (SNVs) are provided in Supplementary Data Set 1.

### Using the molecular consequences of *BRCA* variants to identify distinguishing features

The main purpose of this study was to build an accurate and efficient model that can predict *BRCA1/2* pathogenic variants. Therefore, identifying a set of informative features is crucial for adequate model performance and improving our understanding of the molecular basis of variant pathogenicity.

The final features acquired via greedy feature selection resembled the initial results of the qualitative analysis. To assess how each of the feature categories contributed to the final model, we trained a predictive model using different feature subsets: evolutionary conservation, missense variant prediction models from dbNSFP^28^, physicochemical properties, changes in post-translational modifications.

MCC values representing the performance on the blind test for each subset model were compared (Suppl. Table 3). Noticeably, physicochemical properties WEIL970102 and HENS920101^25^ (MCC = 0.76) were the main contributing features to the final model (*BRCA1/2* combined), followed by other features that contributed to a moderate extent: changes in post-translational modifications^30^ (MCC = 0.75), ClinPred_score and MutationTaster_score^28^(MCC = 0.74).

As a final analysis, we explored the feature importance of the combined *BRCA1/2* model. Suppl. Figure 5 shows that the sequence conservation feature PANTHER^26^ is the most contributing feature followed by PolyphenScore^28^ (a deleterious scoring method). On the other hand, most measures of conservation (SIFT^10^, SNAP2^36^, and Provean^29^) contributed to a moderate extent to the final model.

### Validation of *BRCA1/2* general pathogenicity predictor using Functional Data

To evaluate the robustness of the *BRCA1/2*-general model, several types of functional data reported by Hart^4,11^, Startia^37^, and Findlay^38^ comprising *BRCA1* and *BRCA2* variants and their functional scores (with previously established cut-off points for pathogenic variants) were applied as an independent blind test set to validate our model. The combined experimental functional data contained 2,882 *BRCA1* SNVs from RING and BRCT domains evaluated using different functional assays^4,37,38^ and 229 *BRCA2* SNVs from the DNA binding domain assessed using the HDR assay^4,11^. 2,906 out of the 3,135 *BRCA1/2* variants reported in the previously mentioned studies were not present in our training dataset.

**Figure 4:**
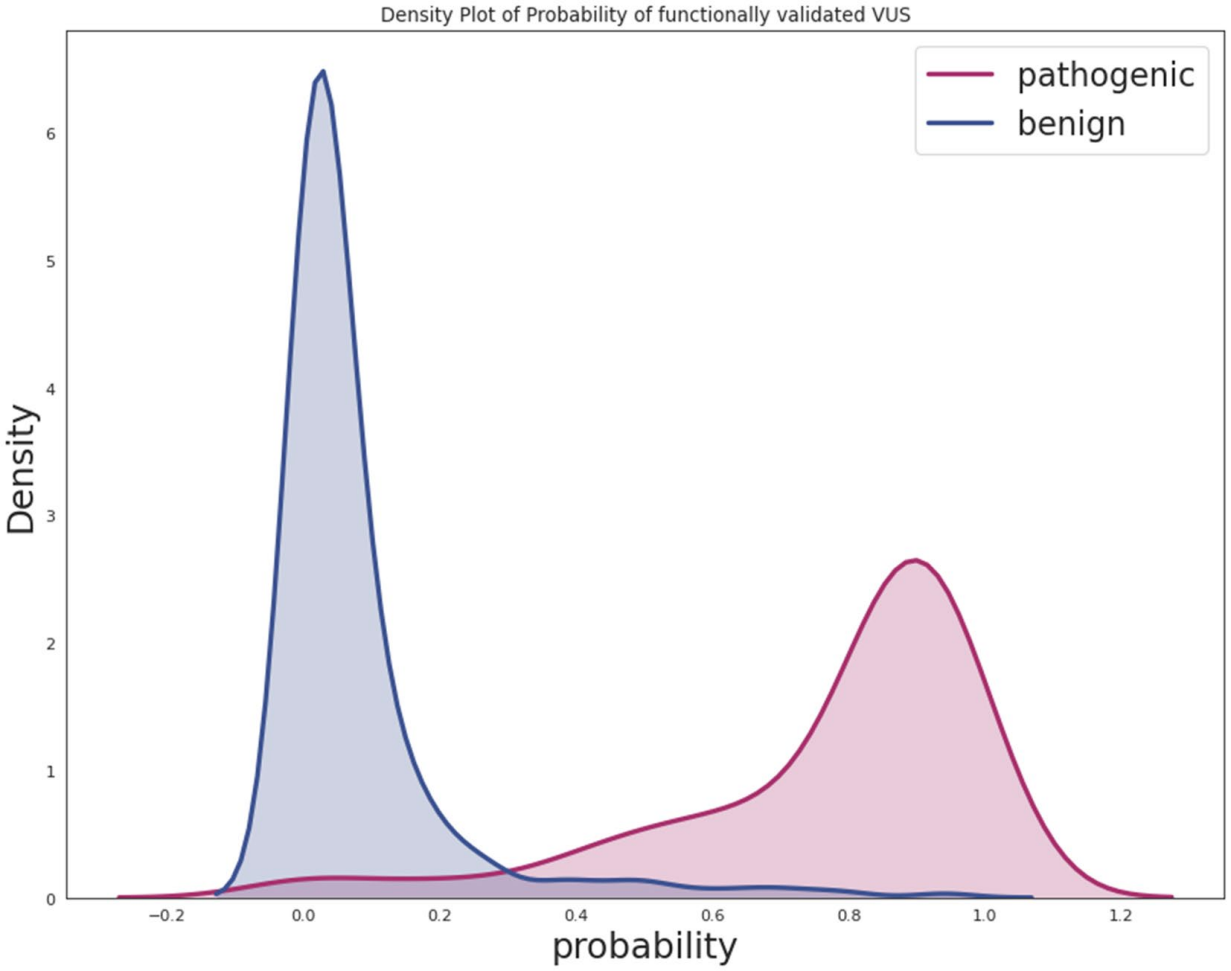
Distribution of probability scores predicted by our final model for functionally assessed VUSs in *BRCA1* and *BRCA2*.

Our model achieved an accuracy of 92% and F1-score of 0.93 for those variants not incorporated in the training data (2,906 variants), highlighting the robustness of our predictive model, and providing confidence in the generalizability of the final model. Figure 4 shows the confidence scores distribution of the functionally evaluated pathogenic and benign VUSs in *BRCA1/2*, demonstrating a good separation between classes.

To showcase the performance of our method, we have assessed two variants. P34L and T1684P are currently classified as VUSs and were predicted as pathogenic at very high probabilities (of 0.88 and 0.91, respectively). Following the present results, a previous study demonstrated that these two variants were designated non-functional, based on functional scores obtained by saturation genome editing functional assay^38^. Furthermore, the P34L and T1684P variants are present in the Ring and BRCT domains of BRCA1, respectively. The P34L variant is predicted to destabilise the structure (-0.84 kcal/mol—mCSM-Stability^39^), with the conversion to Leucine (Leu) altering the backbone structure, leading to loss of rigidity and steric clashes to accommodate Leu (Suppl. Figure 6a). Interestingly, the T1684P variant was also predicted to cause destabilisation of the protein (-0.23 kcal/mol—mCSM-Stability^39^). The proline substitution could disturb the α-helical conformation by intervening intramolecular H-bonding loss of the main-side H-bond and flexibility by eliminating the amide hydrogen required for hydrogen bonding (Suppl. Figure 6b). Suppl. Figure 6 illustrates the interatomic interaction of P34L and T1684P variants.

It was illustrated in a previous study using a multiplex HDR reporter assay that (amino acids 2–96) tended to have the highest proportion of non-functional variants, as the RING domain is encoded almost totally by these positions that are involved in the stability, folding, and function of the full-length protein^37,38^. Additionally, BRCA1 missense variants that are known to predispose to cancer map to either the RING or BRCT domain^37^.

### Comparison with other available methods

We compared the performance of our model (on both cross-validation and blind test sets) with well-established predictors designed to predict the functional effects of missense variants (PolyPhen-2^15^, SIFT^12^ , Align-GVGD^19,20^ , REVEL^13^ and CADD^40^). Additionally, we compared the performance of our models with other available studies ^11,38,41,42^. The comparative prediction performance of the classification models on cross-validation is shown in Table 2. Our models significantly outperformed alternative approaches, with the *BRCA1* model obtaining an accuracy of 0.96 compared to 0.75 for MLR-CAGI^42^, while the BRCA2 model achieved 0.97. Table.3 illustrates the comparative performance of the classification models on blind test sets. Our *BRCA1/2* general model obtained an AUC of 0.96 and 0.95 on cross-validation and blind test sets, respectively, outperforming PolyPhen-2^15^ (0.66,0.77),SIFT^12^ (0.78,0.79) and Align-GVGD^19,20^ (0.53,0.59) , REVEL^13^ (0.79,0.86) and CADD^40^ (0.84,0.79). The predictive models show a significant improvement in the robustness and predictive power compared to previous methods in both data sets (Table2,3).

**Table 2:** Comparative Performance on cross-validation between *BRCA1/2* classification models and other available approaches.

	Methods	ACC	MCC	Sens	Spec	ROC-AUC
*BRCA1*	*BRCA1*-Ring domain^41^	–	–	95%	100%	0.99
	MLR^42^	0.82	0.65	69%	93%	–
	MLR-CAGI^42^	0.75	0.50	61%	87%	–
	SIFT^12^	0.68	0.50	92%	55%	0.73
	PolyPhen-2^15^	0.64	0.40	55%	69%	0.62
	Align-GVGD^19,20^	0.48	0.18	48%	26%	0.58
	REVEL^13^	0.68	0.48	68%	55%	0.74
	CADD^40^	0.74	0.58	74%	61%	0.80
	*BRCA1*	0.96	0.96	93%	98%	0.98
	*BRCA1*-ENIGMA	0.99	0.96	99%	99%	0.98
*BRCA1*-combined	SGE^38^	–	–	96.7%	98.2%	0.98
	BRCA-ML^11^	–	0.66	89.5%	91.5%	–
	*BRCA1*-Ring domain^41^	–	–	97%	83%	0.96
	NN^42^	0.87	0.75	92%	85%	–
	NN-CAGI^42^	0.76	0.55	86%	71%	–
	SIFT^12^	0.68	0.50	92%	55%	0.74
	PolyPhen-2^15^	0.64	0.40	55%	69%	0.62
	Align-GVGD^19,20^	0.40	0.18	38%	23%	0.54
	REVEL^13^	0.68	0.48	68%	55%	0.74
	CADD^40^	0.74	0.58	74%	60%	0.80
	Our model	0.97	0.96	94%	98%	0.99
*BRCA2*	MLR^42^	0.78	0.57	82%	74%	–
	MLR-CAGI^42^	0.86	0.71	86%	85%	–
	SIFT^10^	0.64	0.45	96%	58%	0.77
	PolyPhen-2^13^	0.61	0.43	89%	56%	0.72
	Align-GVGD^19,20^	0.31	0.18	31%	24%	0.47
	REVEL^13^	0.85	0.48	85%	89%	0.77
	CADD^40^	0.76	0.58	76%	71%	0.86
	*BRCA2*	0.98	0.98	96%	98%	0.97
	*BRCA2*-ENIGMA	0.99	0.95	99%	99%	0.99
*BRCA2*-combined	BRCA-ML^9^	–	0.73	97.7%	85.1%	–
	NN^42^	0.84	0.59	75%	86%	–
	NN-CAGI^42^	0.76	0.47	75%	77%	–
	SIFT^10^	0.72	0.45	93%	68%	0.80
	PolyPhen-2^13^	0.68	0.40	88%	64%	0.76
	Align-GVGD^19,20^	0.30	0.18	30%	22%	0.49
	REVEL^13^	0.87	0.54	87%	91%	0.79
	CADD^40^	0.80	0.54	80%	77%	0.85
	Our model	0.91	0.89	82%	93%	0.92
*BRCA1/2* general model	BRCA-ML^9^	–	–	74%	98%	–
	SIFT^10^	0.70	0.48	92%	63%	0.78
	PolyPhen-2^13^	0.66	0.40	66%	66%	0.66
	Align-GVGD^19,20^	0.38	0.06	38%	24%	0.53
	REVEL^13^	0.75	0.50	75%	72%	0.79
	CADD^40^	0.78	0.60	78%	72%	0.84
	Our model	0.96	0.91	96%	98%	0.96

**Table 3:** Comparative Performance on blindtest sets between *BRCA1/2* classification models and other alternative predictors.

	Methods	ACC	MCC	Sens	Spec	ROC-AUC
*BRCA1*	SIFT^10^	0.67	0.62	76%	64%	0.81
	PolyPhen-2^13^	0.68	0.41	68%	59%	0.71
	Align-GVGD^19,20^	0.48	0.18	48%	26%	0.64
	REVEL^13^	0.41	0.18	41%	20%	0.54
	CADD^40^	0.74	0.58	74%	59%	0.80
	Our model	0.86	0.89	92%	82%	0.95
*BRCA1*-combined	SIFT^10^	0.67	0.62	76%	64%	0.81
	PolyPhen-2^13^	0.68	0.41	68%	59%	0.71
	Align-GVGD^19,20^	0.40	0.18	38%	23%	0.64
	REVEL^13^	0.41	0.18	41%	21%	0.54
	CADD^40^	0.74	0.58	73%	59%	0.80
	Our model	0.94	0.89	100%	91%	0.95
*BRCA2*	SIFT^10^	0.62	0.42	62%	52%	0.76
	PolyPhen-2^13^	0.58	0.40	58%	48%	0.74
	Align-GVGD^19,20^	0.39	0.34	38%	24%	0.62
	REVEL^13^	0.84	0.51	85%	86%	0.82
	CADD^40^	0.58	0.58	58%	48%	0.74
	Our model	0.92	0.89	60%	100%	0.98
*BRCA2*-combined	SIFT^10^	0.74	0.51	74%	70%	0.85
	PolyPhen-2^13^	0.69	0.46	69%	64%	0.82
	Align-GVGD^19,20^	0.46	0.28	46%	36%	0.68
	REVEL^13^	0.87	0.61	86%	88%	0.86
	CADD^40^	0.74	0.58	74%	70%	0.85
	Our model	0.97	0.83	86%	100%	0.98
*BRCA1/2*-general model	SIFT^10^	0.69	0.52	69%	59%	0.79
	PolyPhen-2^13^	0.65	0.47	65%	54%	0.77
	Align-GVGD^19,20^	0.44	0.19	44%	30%	0.59
	REVEL^13^	0.82	0.64	82%	78%	0.86
	CADDyyy^40^	0.69	0.58	69%	59%	0.79
	Our model	0.92	0.76	93%	100%	0.95

### Comparison with alternative approaches that rely on genetic data

As in our study we aim at predicting the molecular consequences of *coding* variants *(missense* variants*)* in *BRCA1* and *BRCA2*, we compared the performance of our *BRCA1* and *BRCA2* models with other studies that solely rely on genetic data and likelihood ratios to identify pathogenic variants.

Easton et al.^43^ built a logistic regression model to evaluate the clinical significance of 1,433 sequence variants of unknown significance (VUSs) in *BRCA 1* and *2*, reporting an AUC of 0.80 and 0.70 on their *BRCA1* and *BRCA2* models, respectively. In a similar way, many previous studies (Lindor, 2011^9^; Parsons, 2019)^31^ employed a Multifactorial Probability-Based Model (posterior probability model) for classifying VUSs in *BRCA1* and *BRCA2* that incorporate different forms of genetic evidence. For instance, Parsons et al.^31^ achieved an AUC of 0.78 and an accuracy of 0.80 on their *BRCA1/2* model. In comparison, Lindor et al. (2011)^9^ obtained an AUC of up to 0.93 and an accuracy of up to 0.92 on their *BRCA1* and *BRCA2* models.

Similarly, MS et al*.* (2020)^3^ employed logistic regression to indicate carrier level based on personal and family history of cancer and calculate likelihood ratios denoting pathogenicity. By analysing ~ 138,000 individuals carrying 2,383 *BRCA1/2* variants tested by multigene panel testing (MGPT), their model achieved an AUC of up to 0.83 for *BRCA1* and up to 0.70 for *BRCA2.*

Our models significantly outperformed alternative approaches, *BRCA1* model obtaining an AUC of 0.98 and an accuracy of 0.96, while the *BRCA2* model achieved an AUC of 0.97 and an accuracy of 0.98. The considerably higher performance of our method highlights the necessity to consider protein information to predict pathogenic variants in *BRCA1/2*.

### Comparison of *BRCA1/2*-general predictor with ACMG/AMP classification

To demonstrate the robustness of our final model (*BRCA1/2* general) in classifying VUSs, we compared our final model classification results with the American College of Medical Genetics and Genomics (ACMG) and the Association for Molecular Pa-theology (AMP) scoring^44^, by applying a bioinformatics tool, InterVar^45^.

It was possible to compare most of the *BRCA1/2* missense variants with Intervar^45^. Among the *BRCA1* and *BRCA2* (VUSs) classified as pathogenic by our final model, none were categorised into this class by Intervar^45^. In contrast, the missense variants classified pathogenic were categorised as either likely pathogenic or likely benign by InterVar or remained VUSs.

Noticeably, only ~ 2% of *BRCA1/2* missense variants (VUSs) classified as benign by our final model were categorised as likely pathogenic by Intervar^45^. On the other hand, the prevalence of additional missense variants classified as benign remained VUSs or likely benign with the InterVar tool^45^.

We observed many dissimilarities between our final model prediction and the InterVar tool ACMG/AMP variants scoring. This observance is in line with a recent study^33^ that revealed distinctions between their classification established on a multifactorial model and ACMG/AMP scoring.

## Discussion

Achieving reliable estimations of cancer risk and functional consequence of *BRCA1* and *BRCA2* sequence variants represent a potential to improve management, diagnosis, and clinical decisions of inherited breast and ovarian cancers^38,46^ and computational approaches can enable and support these estimations.

Our study aims to classify and comprehensively estimate the functional consequences of missense variants in *BRCA1/2* genes. We have shown that incorporating machine learning approaches with general pathogenicity scoring systems and mutation physicochemical properties is an effective strategy to obtain accurate predictive models for identifying deleterious missense variants in *BRCA1* and *BRCA2,* which might lead to assisting classification of variants of uncertain significance that currently restrict the interpretation of genomic testing data. The final models obtained for each gene presented statistically significant improvements in comparison with other available approaches.

Wide-scale experimental mutational scanning methods, as in the cases illustrated by Findlay^38^ and Starita^37^ have provided a broader view of the mutational landscape in BRCA1/2. Although these studies functionally classified thousands of variants (1056 and 3893, respectively), there are still over 12,520 and 22,772 possible unclassified missense variants in *BRCA1* and *BRCA2*^9^, that can be investigated efficiently using computational tools.

There are, however, still many limitations to applying these models. The number of experimentally validated deleterious variants in *BRCA1* and *BRCA2*, necessary for model development, is limited, imposing a challenge for machine learning methods and restrains generalization capabilities. In addition, training data are restricted to defined variants that are in protein regions identified to be involved with impaired DNA repair. For instance, the only BRCA2 missense variants, which are known to be disease-causing, are in the DNA-binding domain. It is not understood whether variants located in other domains, which our model predicted, and others predict to be disease-causing, can repress DNA repair.

Nevertheless, the *BRCA1/2* combined model used for predicting the functional impact of all possible missense variants in *BRCA1* and *BRCA2* demonstrated a sensitivity of 96% and 98% specificity, implying that extrapolation beyond the identified domains could be possible. Employing additional pathogenic and neutral measures could determine whether other components of these genes reflect pathogenicity as well as predict their functional impacts.

Here we demonstrate that our final model (*BRCA1/2* combined) is a reliable approach to classify thousands of missense variants in a clinically actionable gene. We anticipate that the *in-silico* saturation mutagenesis methods would become applicable and reliable for interpreting variants of unknown significance, as well as for providing direct functional estimations for newly observed variants. Moreover, the improved performance in our predictive models could assist researchers in prioritising potential SNVs in *BRCA1* and *BRCA2* for further exploration and validation. The results of the computational saturation mutagenesis were made available to researchers (see Supplementary Data Set 1 for all potential SNVs in both genes).

## Methods

### Data sets

To build a gene-specific model as well as a generic model for predicting the functional impact of missense variants in *BRCA1* and *BRCA2*, variants of both genes reviewed by an expert panel (3 stars) and had no conflicting interpretations were curated from the ClinVar^10^ database. In this study, two different datasets were used for each gene to build and train a predictive model, comprising 247 missense variants (pathogenic:97; benign:150) for *BRCA1*, and a total of 189 missense variants (pathogenic:43; benign:146) for *BRCA2* as the primary datasets. Moreover, the benign or likely benign variants retrieved from ClinVar (with no conflicting interpretations) in the combined datasets were grouped into the benign category, and variants interpreted as pathogenic or likely pathogenic were grouped as pathogenic. In comparison, the combined datasets consisted of a total of 335 missense variants for *BRCA1* and a total of 297 missense variants for the *BRCA2* gene.

Furthermore, we have utilised *BRCA1/2* missense variants that ENIGMA^31^ quantitatively and qualitatively classified as pathogenic/benign to increase the reliability of our gene-specific models. The classification of these variants was initially derived based on a multifactorial model and causality scores ranking to assess their clinical significance. We included missense variants if they fulfilled the following standards: pathogenic or benign labels, posterior probability score from multifactorial likelihood analysis ≥ 0.99 (pathogenic) or < 0.99 (benign), or International Agency for Research on Cancer (IARC) class1 (benign) and 5 (pathogenic)^47^. (See Supplementary Data Set 2 for more details on the variants used and analysed in the calculations).

The ENIGMA datasets used comprised 141 missense variants (pathogenic:29; benign:112) for *BRCA1* and a total of 118 missense variants (pathogenic:11; benign:107) for the *BRCA2* gene. The functional validation datasets used in our study were from Hart^4,9^, Starita^37^, and Findlay^38^. Notably, we have only kept the variants that had a functional impact at the protein level, *i.e.*, nonsynonymous missense variants, excluding splicing variants.

All datasets were divided into a training (85%) and blind test (15%) to train and evaluate the predictive/generalisation performance of the predictive models used for the classification task.

### Feature engineering and selection

In this study, a range of features was calculated using different in silico tools to evaluate and predict the molecular and functional consequences of missense variants in *BRCA1* and *BRCA2*.These features incorporated distinct categories, including, evolutionary conservation, protein post-translational modifications (PTMs) changes, sequence properties, biophysical characterization, and variants deleteriousness and pathogenicity evaluation. Supplementary Table 1 summarises the list of investigated features.

1. **Conservation and sequence-based**: We estimated the degree of residue conservation using ConSurf^23^. Substitution matrices (PAMs, BLOSUMs)^48^ and aaindex^25^ were calculated to account for the evolutionary conservation scores and physicochemical attributes of amino acids, respectively. Sequence-based Scores from SAAFEC-SEQ^27^ were measured to evaluate the impacts of single point mutations on protein stability and thermodynamics. Additionally, we applied the Missense Tolerance Ratio (MTR)^49^ to measure the deleteriousness of a missense variant by considering its surrounding regional intolerance.
2. **Protein post-translational modifications (PTMs) changes**: We used the AWESOME ^30^ tool to systematically assess the functional mechanism underlying missense variants and their impact on PTMs that include ubiquitination phosphorylation, glycosylation, methylation, and acetylation.
3. **Biophysical characterization**: The Align-GVGD^19,20^ version applied can be found at http://agvgd.hci.utah.edu/agvgd_input.php, which explicitly classifies missense substitutions into neutral or deleterious by combining the biophysical properties of amino acids and protein multiple sequence alignments and does not incorporate splicing.
4. **Prediction based on Deleteriousness and pathogenicity scoring methods**: Deleteriousness scoring methods from dbNSFP^28^ (Suppl. Table 1) were employed to quantify the deleterious effects of missense variants. We estimated the functional consequences of each variant using pathogenicity-based features SNAP2^36^, PANTHER^26^, SIFT^12^, and Provean^29^.

Selecting the best set of features to train predictive models is known to be a challenging problem. A bottom-up greedy feature selection method was employed to reduce the noise of dimensionality. This approach considers each feature independently and iteratively, keeping only the set of features with the best performance^50^.

### Qualitative analysis

To statistically catalog features that differentiate between the two classes (pathogenic and benign) two-sided Welch sample t-test was carried out on the primary and combined datasets by applying a cutoff *p-*value of < 0.05, employing the *ggsignif* package in Rstudio.

### Machine learning approaches

To obtain predictive classification models, we first evaluated several classification algorithms, including Random Forest, Extremely Randomized Trees, Gradient Boosting, and Adaboost using the implementation available on the Scikit-learn library^51^. The predictive models were trained using stratified tenfold cross-validation and evaluated on non-redundant blind tests.

### Model evaluation metrics

The performance of classification models was evaluated using well-established evaluation metrics, including the Area Under the ROC curve (AUC), Matthew’s Correlation Coefficient (MCC), Precision, F1 Score, Sensitivity, and Specificity. AUC is an effective measure to evaluate the performance of a model in a classification task at various threshold settings. Higher AUC means that the model is robust and capable of distinguishing between the two classes: pathogenic and benign. AUC ranges from 0 and 1. Therefore, a perfect model would have an AUC of 1, and an AUC of 0.5 indicates that the model is a random classifier. MCC is a balanced metric for assessing a classifier’s performance. The MCC returns values that range between − 1 and 1, where total disagreement in predictions is represented as -1, and a coefficient of 1 indicates a perfect prediction. F1 score is the harmonic mean of Precision and Recall (Sensitivity) of a classifier. Precision is the proportion between the correctly classified as positive and all positives. Recall represents the number of correctly predicted positive observations to all positives (pathogenic) in a dataset. Sensitivity (True Positive Rate) and specificity (True Negative Rate) are statistical measures used to estimate the proportion of positive (pathogenic) and negative (benign) classes that are correctly classified, respectively.

## Supplementary Information

Supplementary Information 1.

Supplementary Information 2.
