# Supplementary material for: Understanding and predicting the functional consequences of missense mutations in BRCA1 and BRCA2
Source: Sci Rep. 2022 Jun 21;12:10458. doi: 10.1038/s41598-022-13508-3 (PMC9213547; doi:10.1038/s41598-022-13508-3)
Supplement: Supplementary file 1 — Supplementary Information 1. [file 41598_2022_13508_MOESM1_ESM.pdf]

Supplementary Materials

Understanding and predicting the functional consequences of missense mutations in BRCA1 and BRCA2

Raghad Al-Jarf,[1,2,3], Mengyuan Shen[1,2,3,4], Douglas E.V. Pires[1,2,3,4,*], David B. Ascher[1,2,3,5,*],

[1]Computational Biology and Clinical Informatics, Baker Heart and Diabetes Institute, Melbourne VIC 3004, Australia

[2]Department of Biochemistry and Molecular Biology, University of Melbourne, Melbourne, VIC 3010, Australia

[3]Systems and Computational Biology, Bio21 Institute, University of Melbourne, 30 Flemington Rd, Parkville VIC 3052, Australia

[4]School of Computing and Information Systems, University of Melbourne, Melbourne, VIC 3053, Australia

[5]Department of Biochemistry, University of Cambridge, 80 Tennis Ct Rd, Cambridge CB2 1GA, UK

*To whom correspondence should be addressed D.B.A. Email: david.ascher@unimelb.edu.au. Correspondence may also be addressed to D.E.V.P. Email:douglas.pires@unimelb.edu.au

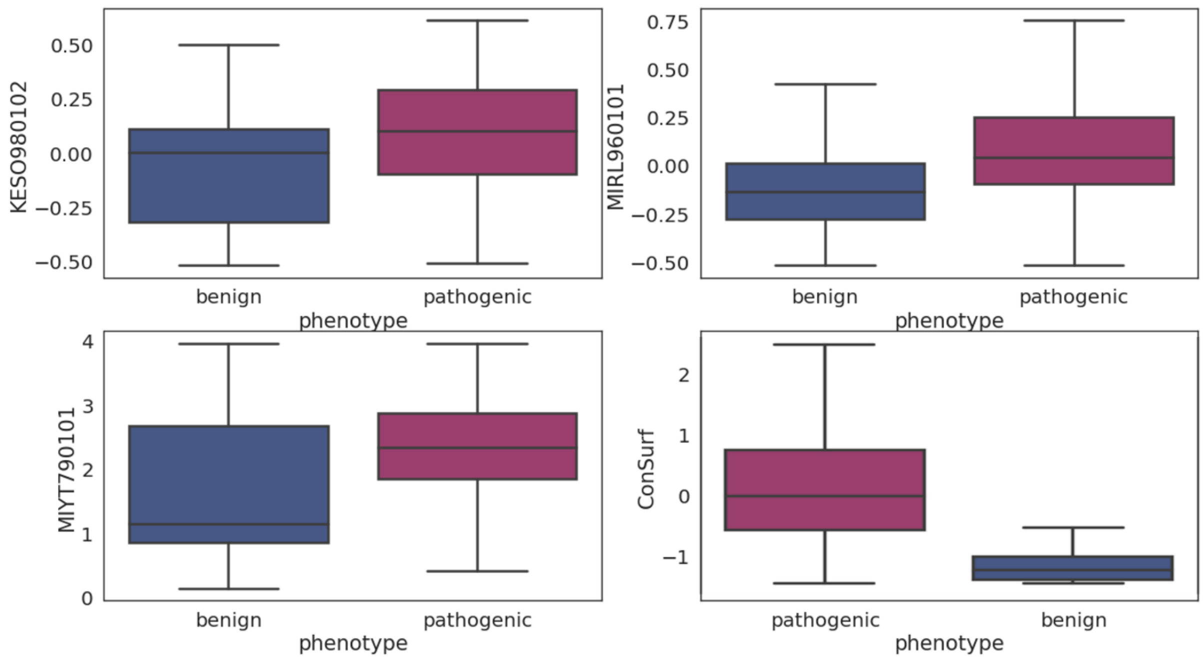

Suppl. Figure 1. The plots depict the top discriminative features of pathogenic in comparison to the benign variants for *BRCA1*, following a Welch sample t-test.

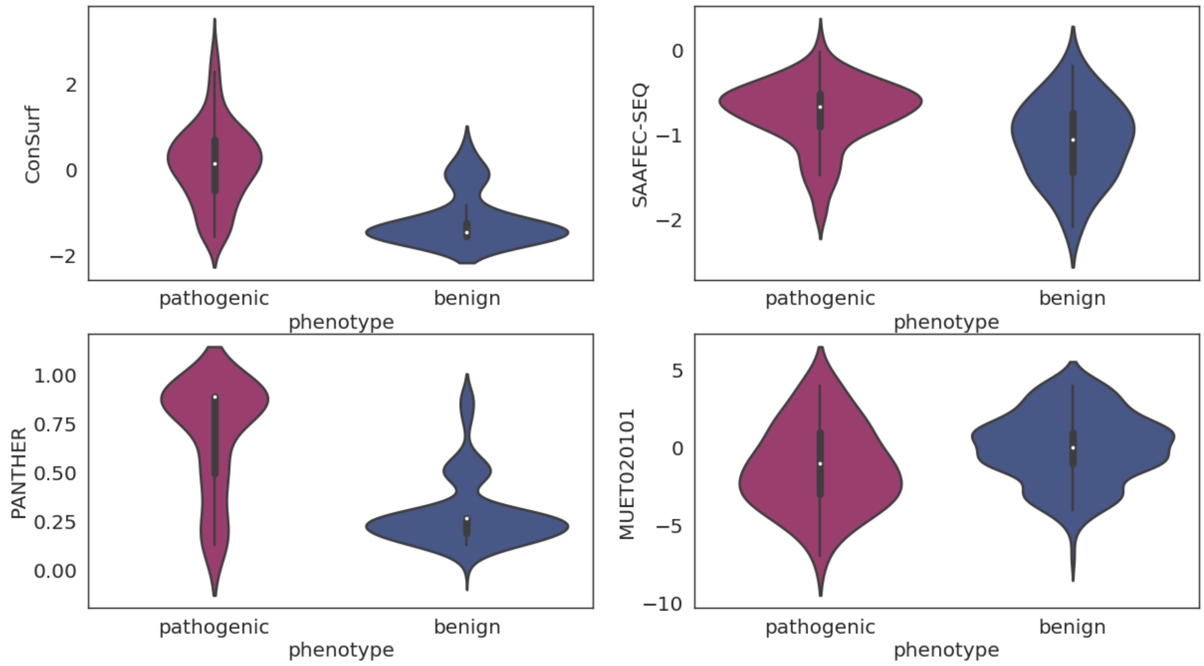

Suppl. Figure 2. The violin plots depict the discriminative properties of pathogenic and benign variants of *BRCA2*, following a Welch sample t-test.

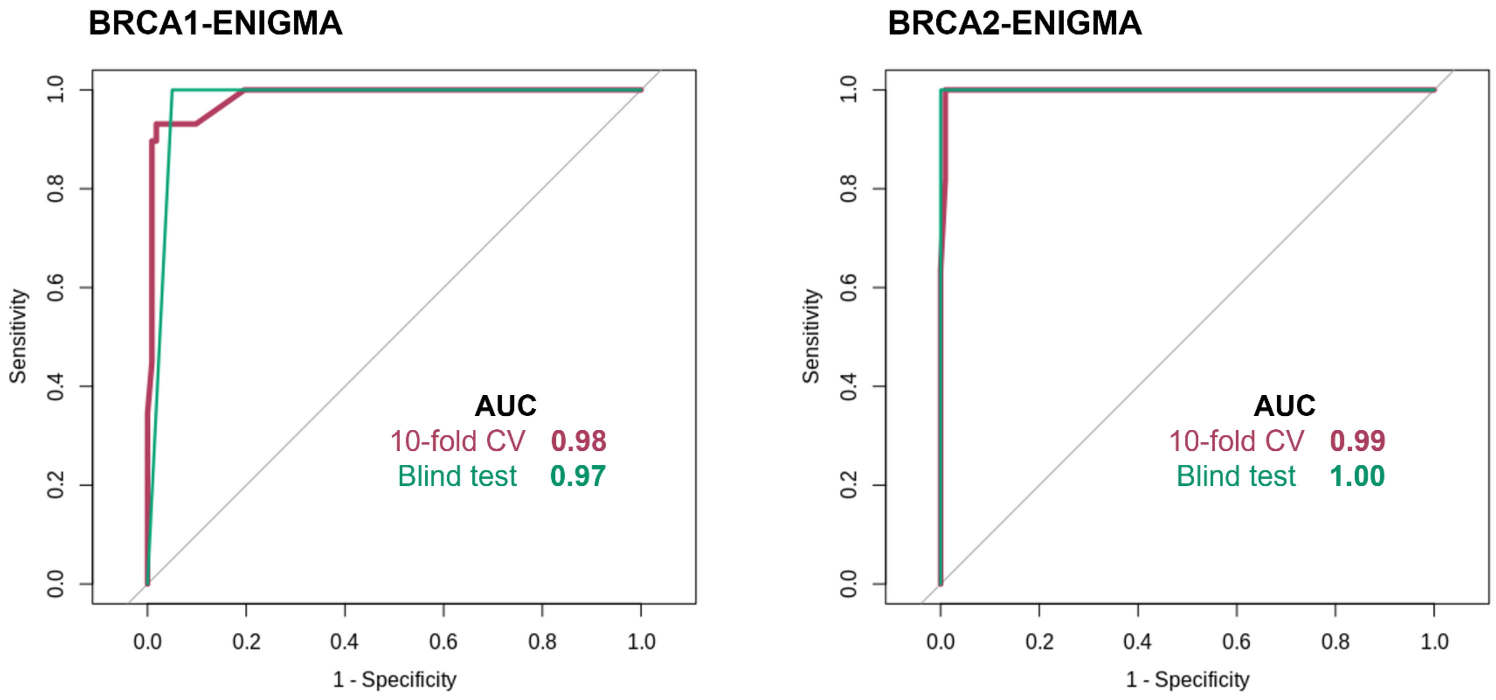

Suppl. Figure 3. Receiver Operating Characteristic (ROC) curve for *BRCA1* (left) and *BRCA2*(right) models on the ENIGMA data. The predictive models accurately identified pathogenic variants with AUC > 0.96 on cross-validation and blind tests.

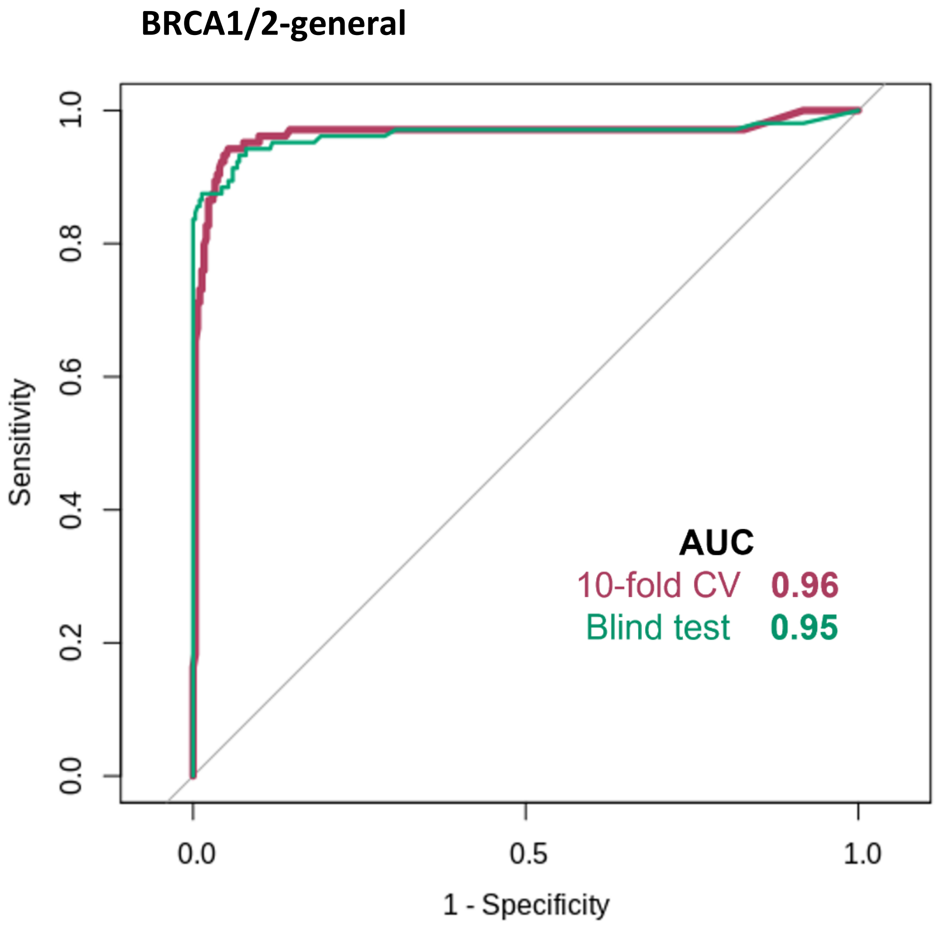

Suppl. Figure 4. Receiver Operating Characteristic (ROC) curve for the final model (*BRCA1/2*-general model). The predictive model accurately identified pathogenic variants with AUC > 0.94 on cross-validation and blind tests.

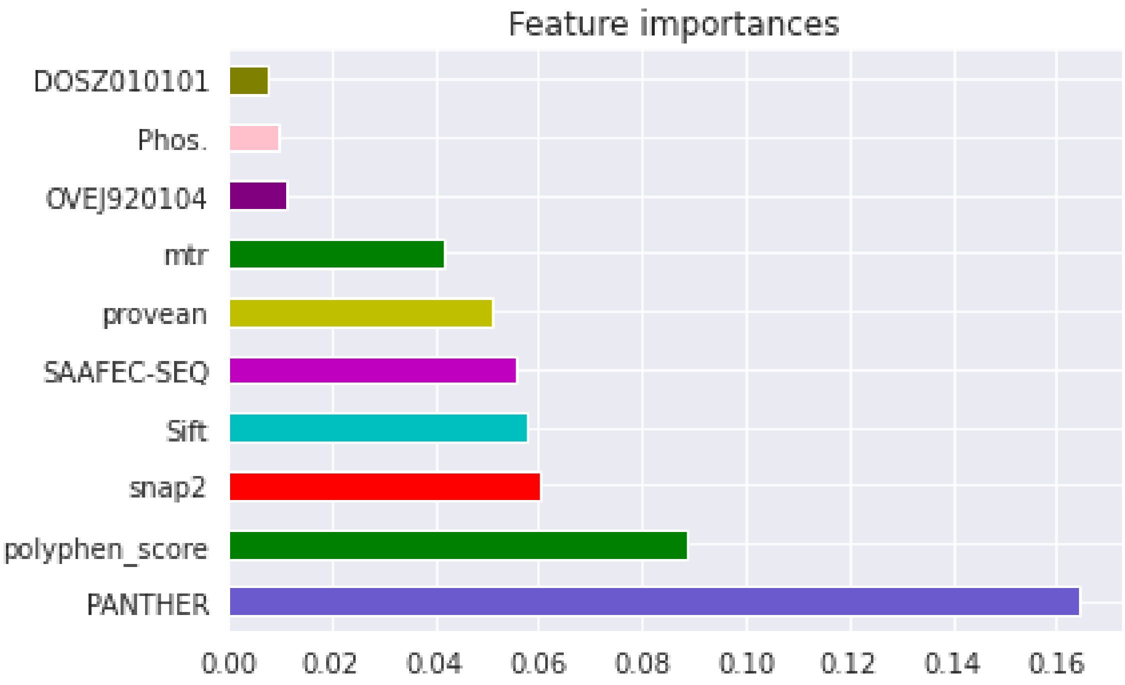

Suppl. Figure 5. Feature importance plot of the input features used to train BRCA1/2 general model.

a.

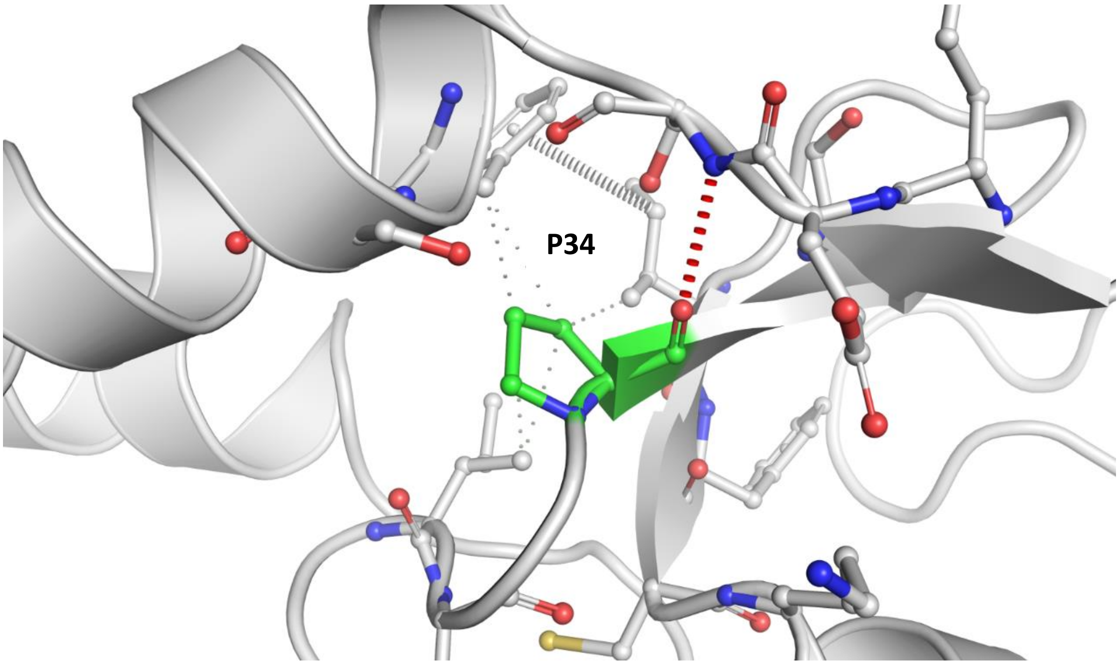

b.

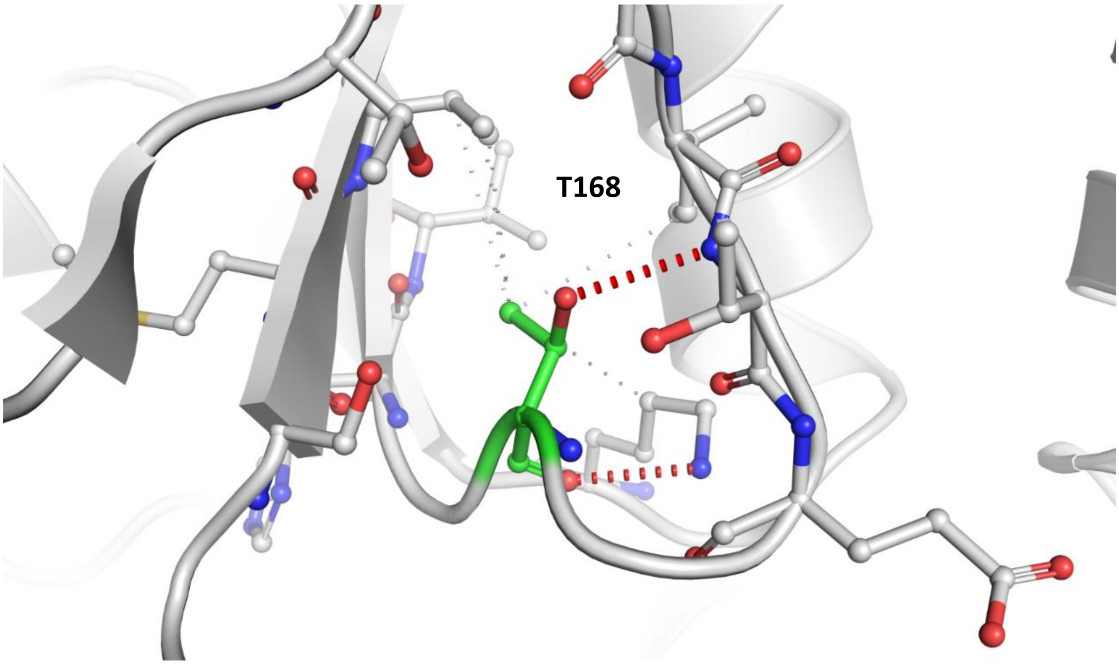

Suppl. Figure 6 illustrations of interatomic interactions at residue P34 (a) and T1684 (b).

Different colours illustrate different types of interactions: for example, halogen bond interactions in blue, hydrophobic in green, and red for hydrogen bonding. The width of each dash indicates the interaction distance; the thickest dashes represent the overlapping van der Waals' radii. The thinnest display interactions that are “proximal” within 5 Å.

Suppl. Table 1. List of the investigated features applied to train *BRCA1* and *BRCA2* classification models.

In silico tool	Feature Category	Feature description
**SNAP2**	pathogenicity scoring methods	Functional effects/ Pathogenicity prediction
**PANTHER**	pathogenicity scoring methods	Functional effects/ Pathogenicity prediction
**SIFT**	pathogenicity scoring methods	Functional effects/ Pathogenicity prediction
**Provean**	pathogenicity scoring methods	Functional effects/ Pathogenicity prediction
**ConSurf**	Conservation	Residue evolution rate
**PAMs**	Conservation	Evolutionary conservation scores
**BLOSUMs**	Conservation	Evolutionary conservation scores
**SAAFEC-SEQ**	Sequence-based properties	Measuring changes in protein Stability and thermodynamics
**MTR**	Sequence-based properties	Missense Tolerance Ratio
**AlignGVGD**	Biophysical characterization	Biochemical/sequence variation
**AWESOME**	Protein post-translational modifications (PTMs) changes	AWESOME score
**Phos (AWESOME tool)**	PTMs changes	Phosphorylation
**Ubi. (AWESOME tool)**	PTMs changes	Ubiquitination
**Meth. (AWESOME tool)**	PTMs changes	Methylation
**SUMO. (AWESOME tool)**	PTMs changes	Sumoylation
**O-GalNAc. (AWESOME tool)**	PTMs changes	O-linked glycosylation,
**O-GlcNAc.(AWESOME tool)**	PTMs changes	O-linked glycosylation,
**N-Gly. (AWESOME tool)**	PTMs changes	N-linked glycosylation
**K-Ace. (AWESOME tool)**	PTMs changes	lysine acetylation
**N-t-Ace. (AWESOME tool)**	PTMs changes	N-terminal acetylation
**Condel**	Deleteriousness scoring methods from dbNSFP	Deleterious functional effects prediction
**CADD_phred**	Deleteriousness scoring methods from dbNSFP	Deleterious functional effects prediction
**ClinPred_rankscore**	Deleteriousness scoring methods from dbNSFP	Deleterious functional effects prediction
**ClinPred_score**	Deleteriousness scoring methods from dbNSFP	Deleterious functional effects prediction
**DANN_rankscore**	Deleteriousness scoring methods from dbNSFP	Deleterious functional effects prediction
**DANN_score**	Deleteriousness scoring methods from dbNSFP	Deleterious functional effects prediction

**Eigen-phred_coding**	Deleteriousness scoring methods from dbNSFP	Deleterious functional effects prediction
**Eigen-raw_coding**	Deleteriousness scoring methods from dbNSFP	Deleterious functional effects prediction
**FATHMM_score**	Deleteriousness scoring methods from dbNSFP	Deleterious functional effects prediction
**GenoCanyon_rankscore**	Deleteriousness scoring methods from dbNSFP	Deleterious functional effects prediction
**GenoCanyon_score**	Deleteriousness scoring methods from dbNSFP	Deleterious functional effects prediction
**M-CAP_rankscore**	Deleteriousness scoring methods from dbNSFP	Deleterious functional effects prediction
**M-CAP_score**	Deleteriousness scoring methods from dbNSFP	Deleterious functional effects prediction
**MPC_rankscore**	Deleteriousness scoring methods from dbNSFP	Deleterious functional effects prediction
**MPC_score**	Deleteriousness scoring methods from dbNSFP	Deleterious functional effects prediction
**MVP_rankscore**	Deleteriousness scoring methods from dbNSFP	Deleterious functional effects prediction
**MetaSVM_rankscore**	Deleteriousness scoring methods from dbNSFP	Deleterious functional effects prediction
**MutPred_rankscore**	Deleteriousness scoring methods from dbNSFP	Deleterious functional effects prediction
**MutPred_score**	Deleteriousness scoring methods from dbNSFP	Deleterious functional effects prediction
**MutationTaster_score**	Deleteriousness scoring methods from dbNSFP	Deleterious functional effects prediction
**Reliability_index**	Deleteriousness scoring methods from dbNSFP	Deleterious functional effects prediction
**REVEL_rankscore**	Deleteriousness scoring methods from dbNSFP	Deleterious functional effects prediction
**REVEL_score**	Deleteriousness scoring methods from dbNSFP	Deleterious functional effects prediction
**PrimateAI_score**	Deleteriousness scoring methods from dbNSFP	Deleterious functional effects prediction
**PrimateAI_rankscore**	Deleteriousness scoring methods from dbNSFP	Deleterious functional effects prediction
**PolyphenScore**	Deleteriousness scoring methods from dbNSFP	Deleterious functional effects prediction
**VEST4_rankscore**	Deleteriousness scoring methods from dbNSFP	Deleterious functional effects prediction
**VEST4_score**	Deleteriousness scoring methods from dbNSFP	Deleterious functional effects prediction
**ALTS910101**	Conservation	Physiochemical properties of amino acids -AA_index
**BENS940101**	Conservation	Physiochemical properties of amino acids -AA_index
**BENS940102**	Conservation	Physiochemical properties of amino acids -AA_index

**BENS940103**	Conservation	Physiochemical properties of amino acids -AA_index
**BENS940104**	Conservation	Physiochemical properties of amino acids -AA_index
**CSEM940101**	Conservation	Physiochemical properties of amino acids -AA_index
**DAYM780301**	Conservation	Physiochemical properties of amino acids -AA_index
**FEND850101**	Conservation	Physiochemical properties of amino acids -AA_index
**FITW660101**	Conservation	Physiochemical properties of amino acids -AA_index
**GEOD900101**	Conservation	Physiochemical properties of amino acids -AA_index
**GONG920101**	Conservation	Physiochemical properties of amino acids -AA_index
**GRAR740104**	Conservation	Physiochemical properties of amino acids -AA_index
**HENS920101**	Conservation	Physiochemical properties of amino acids -AA_index
**HENS920102**	Conservation	Physiochemical properties of amino acids -AA_index
**HENS920103**	Conservation	Physiochemical properties of amino acids -AA_index
**JOHM930101**	Conservation	Physiochemical properties of amino acids -AA_index
**JOND920103**	Conservation	Physiochemical properties of amino acids -AA_index
**JOND940101**	Conservation	Physiochemical properties of amino acids -AA_index
**KOLA920101**	Conservation	Physiochemical properties of amino acids -AA_index
**LEVJ860101**	Conservation	Physiochemical properties of amino acids -AA_index
**LUTR910101**	Conservation	Physiochemical properties of amino acids -AA_index
**LUTR910102**	Conservation	Physiochemical properties of amino acids -AA_index
**LUTR910103**	Conservation	Physiochemical properties of amino acids -AA_index
**LUTR910104**	Conservation	Physiochemical properties of amino acids -AA_index
**LUTR910105**	Conservation	Physiochemical properties of amino acids -AA_index
**LUTR910107**	Conservation	Physiochemical properties of amino acids -AA_index
**MCLA710101**	Conservation	Physiochemical properties of amino acids -AA_index
**MCLA720101**	Conservation	Physiochemical properties of amino acids -AA_index

**MIYS930101**	Conservation	Physiochemical properties of amino acids -AA_index
**MIYT790101**	Conservation	Physiochemical properties of amino acids -AA_index
**MOHR870101**	Conservation	Physiochemical properties of amino acids -AA_index
**NIEK910101**	Conservation	Physiochemical properties of amino acids -AA_index
**NIEK910102**	Conservation	Physiochemical properties of amino acids -AA_index
**OVEJ920101**	Conservation	Physiochemical properties of amino acids -AA_index
**QU_C930101**	Conservation	Physiochemical properties of amino acids -AA_index
**QU_C930102**	Conservation	Physiochemical properties of amino acids -AA_index
**QU_C930103**	Conservation	Physiochemical properties of amino acids -AA_index
**RISJ880101**	Conservation	Physiochemical properties of amino acids -AA_index
**TUDE900101**	Conservation	Physiochemical properties of amino acids -AA_index
**AZAE970101**	Conservation	Physiochemical properties of amino acids -AA_index
**AZAE970102**	Conservation	Physiochemical properties of amino acids -AA_index
**RIER950101**	Conservation	Physiochemical properties of amino acids -AA_index
**WEIL970101**	Conservation	Physiochemical properties of amino acids -AA_index
**WEIL970102**	Conservation	Physiochemical properties of amino acids -AA_index
**MEHP950101**	Conservation	Physiochemical properties of amino acids -AA_index
**MEHP950102**	Conservation	Physiochemical properties of amino acids -AA_index
**MEHP950103**	Conservation	Physiochemical properties of amino acids -AA_index
**KAPO950101**	Conservation	Physiochemical properties of amino acids -AA_index
**VOGG950101**	Conservation	Physiochemical properties of amino acids -AA_index
**KOSJ950101**	Conservation	Physiochemical properties of amino acids -AA_index
**KOSJ950102**	Conservation	Physiochemical properties of amino acids -AA_index
**KOSJ950103**	Conservation	Physiochemical properties of amino acids -AA_index
**KOSJ950104**	Conservation	Physiochemical properties of amino acids -AA_index

**KOSJ950105**	Conservation	Physiochemical properties of amino acids -AA_index
**KOSJ950106**	Conservation	Physiochemical properties of amino acids -AA_index
**KOSJ950107**	Conservation	Physiochemical properties of amino acids -AA_index
**KOSJ950108**	Conservation	Physiochemical properties of amino acids -AA_index
**KOSJ950109**	Conservation	Physiochemical properties of amino acids -AA_index
**KOSJ950110**	Conservation	Physiochemical properties of amino acids -AA_index
**KOSJ950111**	Conservation	Physiochemical properties of amino acids -AA_index
**KOSJ950112**	Conservation	Physiochemical properties of amino acids -AA_index
**KOSJ950113**	Conservation	Physiochemical properties of amino acids -AA_index
**KOSJ950114**	Conservation	Physiochemical properties of amino acids -AA_index
**KOSJ950115**	Conservation	Physiochemical properties of amino acids -AA_index
**OVEJ920102**	Conservation	Physiochemical properties of amino acids -AA_index
**OVEJ920103**	Conservation	Physiochemical properties of amino acids -AA_index
**OVEJ920104**	Conservation	Physiochemical properties of amino acids -AA_index
**OVEJ920105**	Conservation	Physiochemical properties of amino acids -AA_index
**LINK010101**	Conservation	Physiochemical properties of amino acids -AA_index
**BLAJ010101**	Conservation	Physiochemical properties of amino acids -AA_index
**PRLA000101**	Conservation	Physiochemical properties of amino acids -AA_index
**PRLA000102**	Conservation	Physiochemical properties of amino acids -AA_index
**DOSZ010101**	Conservation	Physiochemical properties of amino acids -AA_index
**DOSZ010102**	Conservation	Physiochemical properties of amino acids -AA_index
**DOSZ010103**	Conservation	Physiochemical properties of amino acids -AA_index
**DOSZ010104**	Conservation	Physiochemical properties of amino acids -AA_index
**GIAG010101**	Conservation	Physiochemical properties of amino acids -AA_index
**DAYM780302**	Conservation	Physiochemical properties of amino acids -AA_index

**HENS920104**	Conservation	Physiochemical properties of amino acids -AA_index
**QUIB020101**	Conservation	Physiochemical properties of amino acids -AA_index
**NAOD960101**	Conservation	Physiochemical properties of amino acids -AA_index
**RUSR970101**	Conservation	Physiochemical properties of amino acids -AA_index
**RUSR970102**	Conservation	Physiochemical properties of amino acids -AA_index
**RUSR970103**	Conservation	Physiochemical properties of amino acids -AA_index
**OGAK980101**	Conservation	Physiochemical properties of amino acids -AA_index
**KANM000101**	Conservation	Physiochemical properties of amino acids -AA_index
**NGPC000101**	Conservation	Physiochemical properties of amino acids -AA_index
**MUET010101**	Conservation	Physiochemical properties of amino acids -AA_index
**MUET020101**	Conservation	Physiochemical properties of amino acids -AA_index
**MUET020102**	Conservation	Physiochemical properties of amino acids -AA_index
**TANS760101**	Conservation	Physiochemical properties of amino acids -AA_index
**TANS760102**	Conservation	Physiochemical properties of amino acids -AA_index
**ROBB790102**	Conservation	Physiochemical properties of amino acids -AA_index
**BRYS930101**	Conservation	Physiochemical properties of amino acids -AA_index
**THOP960101**	Conservation	Physiochemical properties of amino acids -AA_index
**MIRL960101**	Conservation	Physiochemical properties of amino acids -AA_index
**VENM980101**	Conservation	Physiochemical properties of amino acids -AA_index
**BASU010101**	Conservation	Physiochemical properties of amino acids -AA_index
**MIYS850102**	Conservation	Physiochemical properties of amino acids -AA_index
**MIYS850103**	Conservation	Physiochemical properties of amino acids -AA_index
**MIYS960101**	Conservation	Physiochemical properties of amino acids -AA_index
**MIYS960102**	Conservation	Physiochemical properties of amino acids -AA_index
**MIYS960103**	Conservation	Physiochemical properties of amino acids -AA_index

**MIYS990106**	Conservation	Physiochemical properties of amino acids -AA_index
**MIYS990107**	Conservation	Physiochemical properties of amino acids -AA_index
**LIWA970101**	Conservation	Physiochemical properties of amino acids -AA_index
**KESO980101**	Conservation	Physiochemical properties of amino acids -AA_index
**KESO980102**	Conservation	Physiochemical properties of amino acids -AA_index
**MOOG990101**	Conservation	Physiochemical properties of amino acids -AA_index
**BETM990101**	Conservation	Physiochemical properties of amino acids -AA_index
**TOBD000101**	Conservation	Physiochemical properties of amino acids -AA_index
**TOBD000102**	Conservation	Physiochemical properties of amino acids -AA_index
**PARB960101**	Conservation	Physiochemical properties of amino acids -AA_index
**PARB960102**	Conservation	Physiochemical properties of amino acids -AA_index
**KOLA930101**	Conservation	Physiochemical properties of amino acids -AA_index
**GODA950101**	Conservation	Physiochemical properties of amino acids -AA_index
**SKOJ970101**	Conservation	Physiochemical properties of amino acids -AA_index
**SKOJ000101**	Conservation	Physiochemical properties of amino acids -AA_index
**SKOJ000102**	Conservation	Physiochemical properties of amino acids -AA_index
**BONM030101**	Conservation	Physiochemical properties of amino acids -AA_index
**BONM030102**	Conservation	Physiochemical properties of amino acids -AA_index
**BONM030103**	Conservation	Physiochemical properties of amino acids -AA_index
**BONM030104**	Conservation	Physiochemical properties of amino acids -AA_index
**BONM030105**	Conservation	Physiochemical properties of amino acids -AA_index
**BONM030106**	Conservation	Physiochemical properties of amino acids -AA_index
**MICC010101**	Conservation	Physiochemical properties of amino acids -AA_index
**SIMK990101**	Conservation	Physiochemical properties of amino acids -AA_index
**SIMK990102**	Conservation	Physiochemical properties of amino acids -AA_index

**SIMK990103**	Conservation	Physiochemical properties of amino acids -AA_index
**SIMK990104**	Conservation	Physiochemical properties of amino acids -AA_index
**SIMK990105**	Conservation	Physiochemical properties of amino acids -AA_index
**ZHAC000101**	Conservation	Physiochemical properties of amino acids -AA_index
**ZHAC000102**	Conservation	Physiochemical properties of amino acids -AA_index
**ZHAC000103**	Conservation	Physiochemical properties of amino acids -AA_index
**ZHAC000104**	Conservation	Physiochemical properties of amino acids -AA_index
**ZHAC000105**	Conservation	Physiochemical properties of amino acids -AA_index
**ZHAC000106**	Conservation	Physiochemical properties of amino acids -AA_index

Suppl. Table 2. The list of the selected features following greedy features selection employed in the *BRCA1/2*-combined final model.

Name of Features	Mean Benign	Mean Pathogenic	P-value*
ClinPred_score	0.5226646	0.9082761	< 2.2e-16
Provean	-0.9022615	-1.1671088	1.353e-11
MutationTaster_score	0.8723180	0.9631856	< 2.2e-16
Ubiquitination	-0.01001467	-0.02878741	0.2639
MIYS990107	-0.007229176	-0.027329932	0.01016
THOP960101	-0.1157790	-0.1388435	0.2702
PolyphenScore	0.3688999	0.6008793	< 2.2e-16
LUTR910107	-0.6534312	1.3333333	2.971e-06
acetylation	0	0	NA
HENS920101	-0.5826500	-0.3044218	0.0003191
WEIL970102	0.3051360	0.2636054	0.4735
MetaSVM_score	0.3173729	0.6563452	< 2.2e-16
MPC_rankscore	0.3173729	0.6563452	< 2.2e-16
WEIL970101	0.2188095	0.3407311	< 2.2e-17
AWESOME_Score	-0.3802331	-0.1462585	9.04e-06

***p-value measured by applying Welch two-sample t-test.**

Suppl. Table 3. Performance on the blind test for subsets of attributes used to build the Final model of *BRCA1/2* -combined.

Name of Features	MCC
**ClinPred_score**	0.742
**MutationTaster_score**	0.742
**Provean**	0.755
**Ubiquitination**	0.755
**MIYS990107**	0.755
**THOP960101**	0.755
**PolyphenScore**	0.755
**LUTR910107**	0.755
**acetylation**	0.755
**MetaSVM_score**	0.755
**MPC_rankscore**	0.755
**AWESOME_Score**	0.755
**WEIL970101**	0.762
**WEIL970102**	0.762
**HENS920101**	0.762
